# Ultrawide-Field Optical Coherence Tomography Angiography-Guided Navigated Laser Therapy of Non-Perfused Areas in Branch Retinal Vein Occlusion

**DOI:** 10.3390/jcm14145014

**Published:** 2025-07-15

**Authors:** Yao Zhou, Peng Peng, Jiaojiao Wei, Jian Yu, Min Wang

**Affiliations:** 1 Eye Institute and Department of Ophthalmology, Eye & ENT Hospital, Fudan University, Shanghai 200031, China; yao.zhou@fdeent.org (Y.Z.); 23211260008@m.fudan.edu.cn (P.P.); 14301050272@fudan.edu.cn (J.W.); 2 NHC Key Laboratory of Myopia and Related Eye Diseases, Key Laboratory of Myopia and Related Eye Diseases, Chinese Academy of Medical Sciences, Shanghai 200031, China; 3 Shanghai Key Laboratory of Visual Impairment and Restoration, Shanghai 200031, China

**Keywords:** UWF-OCTA, BRVO, navigated laser therapy

## Abstract

**Background/Objectives**: This study evaluates whether ultrawide-field optical coherence tomography angiography (UWF-OCTA) can guide navigated laser therapy for non-perfused areas (NPAs) in branch retinal vein occlusion (BRVO). It further explores whether the laser spots can be accurately placed according to plan, considering that the retina is three-dimensional (3D), while UWF-OCTA provides two-dimensional (2D) images. **Methods**: UWF-OCTA images from three devices—VG200, Xephilio OCT-S1, and Bmizar—guided the treatments. These images were superimposed onto NAVILAS^®^ system images to guide NPA treatments. Pre-treatment planning was strategically designed to avoid normal and collateral vessels, with immediate post-laser OCTA and en face images assessing the efficacy of the laser spots in avoiding these vessels as planned. The accuracy of navigated laser therapy was further analyzed by comparing the intended laser locations with the actual spots. **Results**: All montaged OCTA images from the three devices were seamlessly integrated into the navigated laser system without registration errors. All patients received treatments targeting the NPAs as planned. However, not all collateral or normal vessels were successfully avoided by the laser spots. A further analysis revealed that the actual locations of the laser spots deviated slightly from the planned locations, particularly in the mid-periphery areas. **Conclusions**: UWF-OCTA-guided navigated laser photocoagulation is feasible and precise for treating NPAs in BRVO. Nonetheless, minor deviations between planned and actual locations were observed. This discrepancy, particularly important when treating diseases of the macular area, should be carefully considered when employing OCTA-guided navigated laser photocoagulation.

## 1. Introduction

Branch retinal vein occlusion (BRVO) is characterized by focal occlusion of a major retinal vein and often occurs at arteriovenous crossings [1,2]. Macular edema (ME) is the primary cause of vision loss in patients with BRVO. Anti-vascular endothelial growth factor (VEGF) therapy is regarded as the gold-standard treatment for ME secondary to BRVO. However, the clinical challenge of recurrent ME necessitates frequent injections of anti-VEGF agents to avoid recurrence [3,4]. Repeated intravitreal injections increase the risk of adverse events, including endophthalmitis and retinal detachment. Consequently, identifying strategies to minimize the recurrence of ME following intravitreal anti-VEGF therapy is particularly important.

Prior to the development of anti-VEGF therapy, laser photocoagulation was used to reduce VEGF expression by attenuating the oxygen demand in the ischemic retina. However, this approach has been shown to significantly worsen the peripheral visual fields compared with untreated eyes [5]. Therefore, in 1986, the Branch Vein Occlusion Study (BVOS) suggested that photocoagulation treatment should only be performed if neovascularization is present [5,6]. Recent studies have shown that the retinal photocoagulation of peripheral non-perfused areas (NPAs) could prevent the recurrence of ME due to BRVO following intravitreal anti-VEGF drug injections [7,8,9]. It was also shown that the region with reduced visual field sensitivity is strongly associated with NPAs [10], suggesting that accurate laser photocoagulation of NPAs may minimize the effects of the procedure on the peripheral visual fields. Additionally, collateral vessels are often present in eyes with BRVO and might contribute to the absorption of ME [11,12]. Therefore, finding a way to improve the accuracy of the photocoagulation of NPAs without injuring collateral vessels could maximize the effectiveness and reduce the side effects of laser photocoagulation.

A novel laser device comprising a retinal navigation system, the NAVILAS^®^ (OD-OS GmbH, Teltow, Germany), has recently been developed. It incorporates a digital fundus imaging system for live red-free, infrared, and fluorescein angiography (FA) imaging, which is useful for treatment planning. Although FA can create images of the retinal vasculature with good resolution, it cannot directly visualize the capillary network [13]. Compared with FA, optical coherence tomography angiography (OCTA) provides images of retinal capillaries at a relatively high axial resolution without causing dye leakage. Furthermore, OCTA can detect NPAs and collateral vessels in BRVO with greater accuracy than FA [14,15]. Although conventional OCTA can provide 3 × 3 mm to 12 × 12 mm scans, its approximate field of view (FOV) is 56°, which is restricted to the posterior pole. With recent advances in technology, ultrawide-field OCTA (UWF-OCTA) can cover an FOV of 100°−120° and simultaneously capture the posterior pole and the mid-periphery [16,17].

UWF-OCTA has become a valuable tool in evaluating retinal vascular diseases, including diabetic retinopathy (DR) and RVO, offering several advantages over conventional imaging modalities such as standard-field FA or OCTA. Studies showed that vascular diseases can be accurately assessed with WF-OCTA, providing results that surpass those obtained with standard-field FA, with minor effects on comfort, time, and image quality [18,19]. Additionally, OCTA offers depth-resolved angiographic images, allowing for a quantitative analysis of choroidal sublayers, which are often neglected in FA [20]. These results demonstrate the promising prognostic insights and the potential of UWF-OCTA to improve RVO management strategies.

Several studies employing NAVILAS^®^ for laser therapy for proliferative diabetic retinopathy (PDR), retinal hemangioblastomas, and central serous chorioretinopathy (CSC) have demonstrated that navigated laser therapy enhanced precision and safety, suggesting significant potential for optimizing clinical decision-making [21,22,23]. Nevertheless, the application of UWF-OCTA for navigated laser therapy remains unexplored, especially with no existing reports addressing peripheral retinal targeting accuracy. Based on these studies, we hypothesized that UWF-OCTA might be used to guide a navigated laser photocoagulator (NAVILAS^®^) in the treatment of BRVO. However, OCTA images are limited to two dimensions (2D), and the eye fundus is three-dimensional (3D), with a relatively flat posterior region and a steep curvature in the mid-periphery and periphery, which may distort the OCTA image superimposed onto the fundus image. Although Amoroso et al. [24] successfully used OCTA-guided navigated laser therapy for advanced macula neovascularization secondary to age-related macular degeneration, their study was restricted to patients who underwent treatment in the relatively flat posterior region (6 × 6 mm). It remains to be determined whether UWF-OCTA images can be used to guide navigated laser therapy, considering that the steep retinal curvature of the mid-periphery could increase registration errors when attempting to overlay the 2D UWF-OCTA image onto the 3D image of the fundus.

Therefore, the aim of this study was to evaluate the accuracy of UWF-OCTA images for guiding a navigated laser photocoagulator (NAVILAS^®^) for the treatment of NPAs in eyes with BRVO without injuring the collateral vessels.

## 2. Materials and Methods

### 2.1. Subjects

This is a retrospective study that included 28 patients (28 eyes) with BRVO who visited the Eye and ENT Hospital of Fudan University (Shanghai, China) between May 2021 and May 2023. This study was approved by the institutional review board of the Eye and ENT Hospital of Fudan University and adhered to the tenets of the Declaration of Helsinki. All subjects provided written informed consent to undergo laser therapy. Due to the retrospective nature of this study, there was no control or comparator group.

All subjects underwent comprehensive ophthalmic examination, which included evaluations of distance best-corrected visual acuity, measurement of intraocular pressure using a non-contact tonometer, slit-lamp biomicroscopy of the anterior and posterior segments, dilated ophthalmoscope, color fundus photography, and FA prior to laser photocoagulation. UWF-OCTA and UWF fundus photographs were taken before and after laser therapy. The decision regarding the need for laser therapy for BRVO was made by the senior physician (W.M.), in accordance with the guidelines for laser treatment of BRVO [25].

Patients were excluded from this study if they presented with (1) cataract or other media opacity that reduced the quality of fundus imaging; (2) concurrent retinal disease that may result in NPA, such as diabetic retinopathy; (3) conditions precluding laser therapy completion; or (4) extensive hemorrhage that complicated the assessment of NPAs via OCTA.

### 2.2. Image Acquisition Protocol

Patients were randomly assigned to obtain UWF-OCTA images using one of three different instruments: VG200 (SVision Imaging Limited, Luoyang, China), Xephilio OCT-S1 (Canon, Tokyo, Japan), and Bmizar (Toward Pi Medical Technology Limited, Beijing, China). These instruments are equipped with swept-source lasers, operating at central wavelengths of 1050–1060 nm and scan rates of 100,000–400,000 A-scans per second. Initially, for the VG200, montages were created from 12 × 12 mm OCTA scans at five separate eye positions, forming an image of approximately 23.5 × 17.5 mm, equivalent to an FOV of 115°. The Xephilio OCT-S1 and Bmizar devices capture 23–24 × 20 mm OCTA images in a single shot using a special lens. By employing montage techniques, images from the Xephilio OCT-S1 and Bmizar, covering various visual fixations, can expand the FOV to 200°. The 12 × 12 mm and 23–24 × 20 mm OCT images were transferred to and superimposed onto images obtained by the NAVILAS^®^ navigated laser system.

### 2.3. Laser Therapy

Laser photocoagulation was performed using the NAVILAS^®^ laser system (532 nm double-pulsed yttrium aluminum garnet [YAG] laser; OD-OS GMBH, Teltow, Germany). Yellow-wavelength (577 nm) photocoagulation was planned to target the NPAs. Standardized laser parameters were applied across all cases, with adjustments based on the response to the laser treatment: spot size 150–390 μm, duration 100 ms, and power titrated between 100 and 280 mW to achieve mild-gray retinal burn intensity. As the basis of the treatment plan, color images of the retina and infrared images were captured. The selected UWF-OCTA images depicting the NPAs were imported and automatically or manually superimposed onto the captured images. The laser treatment plan aimed to target these NPAs while minimizing the risk of damage to collateral vessels and normal retinal tissue. Treatment plans, prepared by a physician (W.M.), were placed onto the fundus images as a live overlay and stabilized against eye movements during therapy. After photocoagulation, a color image of the fundus was acquired to confirm that all laser applications accurately hit the preplanned points.

Immediately after the laser treatment, another OCTA scan was performed. To compare the actual spot locations with the planned locations, the OCTA images and the en face images obtained before and after laser therapy were carefully merged using Photoshop, based on the optic disc and the large vessels. The layer opacity was adjusted to clearly depict the vessels and the laser spots.

### 2.4. Statistical Analysis

The χ^2^ test was used to compare the success rate of UWF-OCTA images obtained using different devices to guide navigated laser treatment of NPAs. A value of *p* < 0.05 was considered statistically significant.

## 3. Results

### 3.1. Image Registration and Transfer

**Figure 1:**
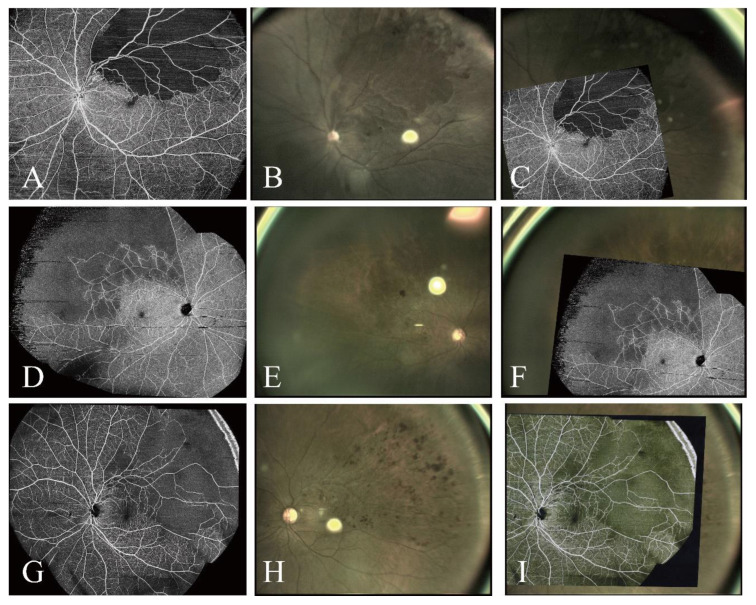
Images from different UWF-OCTA devices can be registered and transferred, demonstrating the interoperability and flexibility of these systems in clinical settings. (**B**,**E**,**H**) Live fundus images captured using the NAVILAS^®^ system. (**A**,**D**,**G**) Montaged OCTA images of 12 × 12 mm fields from five visual fixation scans (**A**) and montaged OCTA images of a 23 × 20 mm scan captured by Xephilio OCT-S1 (**D**) and Bmizar (**G**) devices were successfully imported into the navigated laser system without any registration errors. (**C**,**F**,**I**) OCTA images captured using different UWF-OCTA devices and superimposed on corresponding live fundus images captured using the NAVILAS^®^ system.

Image registration and transfer are two essential components of OCTA-guided navigated laser therapy. Image registration involves aligning the images acquired by various modalities into a unified coordinate system. This process is crucial for surgical planning and navigation because it integrates images bearing vital complementary structural and functional information. The OCTA images were imported into the navigated laser system and manually superimposed on the live fundus images to create the treatment plan for each patient. The montaged OCTA images of 12 × 12 mm fields from five visual fixation scans (Figure 1A–C) and the montaged OCTA images of one 23 × 20 mm scan (Figure 1D–F) were successfully imported into the navigated laser system without registration errors, and there was no difference in transmission speed and image quality.

### 3.2. Therapy of NPAs

**Figure 2:**
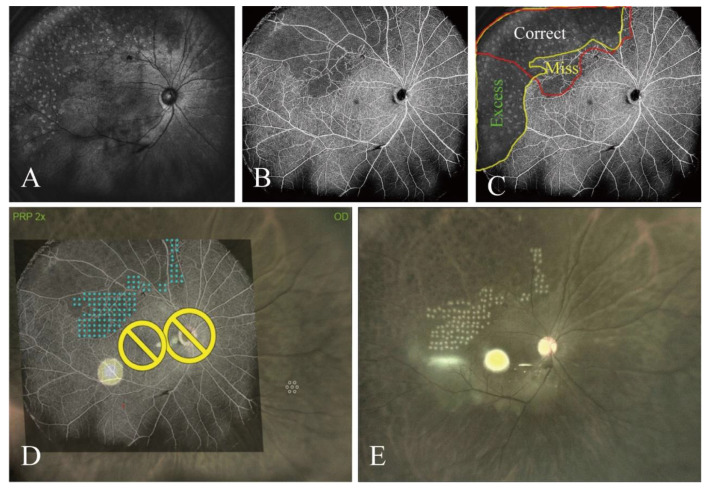
UWF-OCTA-guided navigated laser therapy successfully treated areas missed by conventional pattern laser photocoagulation. (**A**) En face OCT image showing spots after conventional pattern laser photocoagulation. (**B**) OCTA image showing the NPA. (**C**) The OCTA image overlaid on the en face OCT image, showing that conventional pattern laser photocoagulation cannot treat NPAs precisely because of inadequate and excess laser spots. (**D**) UWF-OCTA-guided navigated laser (blue spots) plan to target the inadequately treated areas. (**E**) Live fundus image after navigated laser treatment shows the areas were treated correctly. Blue spots indicate planned laser spots, yellow circle represents optic disc and macula avoided during laser treatment.

**Figure 3:**
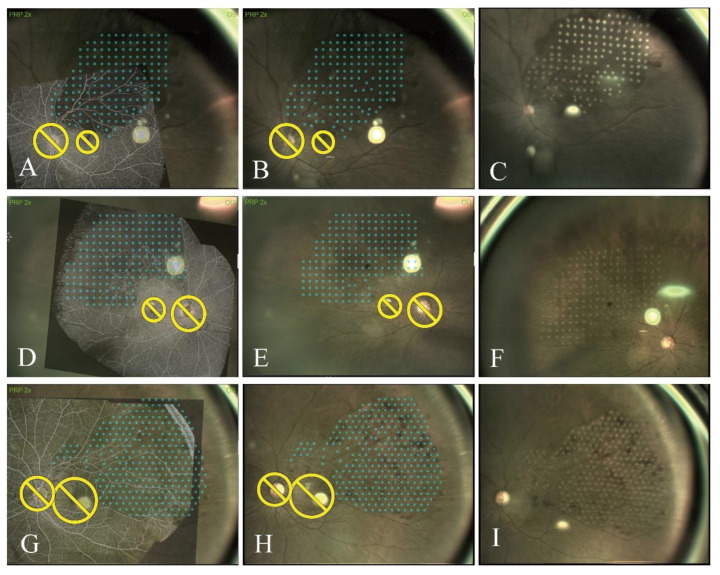
Different UWF-OCTA devices can be used to guide navigated laser photocoagulation for the treatment of NPAs in patients with untreated BRVO. (**A**–**C**) OCTA image that is a montage of five OCT images (12 × 12 mm) captured with a VG200 and superimposed on corresponding live fundus images, used for treatment plans. After photocoagulation, a color image of the fundus was captured. (**D**–**F**) OCTA image that is a montage of two OCT images (23 × 20 mm) captured with a Xephilio OCT-S and superimposed on corresponding live fundus images, used for treatment plans. After photocoagulation, a color image of the fundus was captured. (**G**–**I**) OCTA image that is a montage of two OCT images (24 × 20 mm) captured with a Bmizar and superimposed on corresponding live fundus images, used for treatment plans. After photocoagulation, a color image of the fundus was captured. Yellow circles represents optic disc and macula avoided during laser treatment. Blue spots indicate planned laser spots.

Conventional pattern laser photocoagulation, which is dependent on the operator’s experience, fails to accurately identify NPAs (Figure 2A–C) following conventional treatment. The post-BRVO OCTA and en face OCT images show that this technique cannot target NPAs precisely. Furthermore, inadequate and excess laser spots were observed in the retina (Figure 2C). This patient experienced recurrent ME after repeated intravitreal injections of an anti-VEGF therapy. Consequently, UWF-OCTA-guided navigated laser was performed to target the areas missed in the previous treatment (Figure 2D). As shown in Figure 2E, the areas missed previously were successfully treated in accordance with the treatment plan. Figure 3 shows examples of images obtained by different UWF-OCTA devices used to guide navigated laser therapy of NPAs in patients with untreated BRVO. Navigation was performed using UWF-OCTA images from a VG200 in ten patients, from a Xephilio OCT-S1 in ten patients, and from a Bmizar in eight patients. All 28 patients underwent treatment targeting the NPAs, as predefined (Table 1).

### 3.3. Protecting Collateral Circulation or Normal Vessels

**Figure 4:**
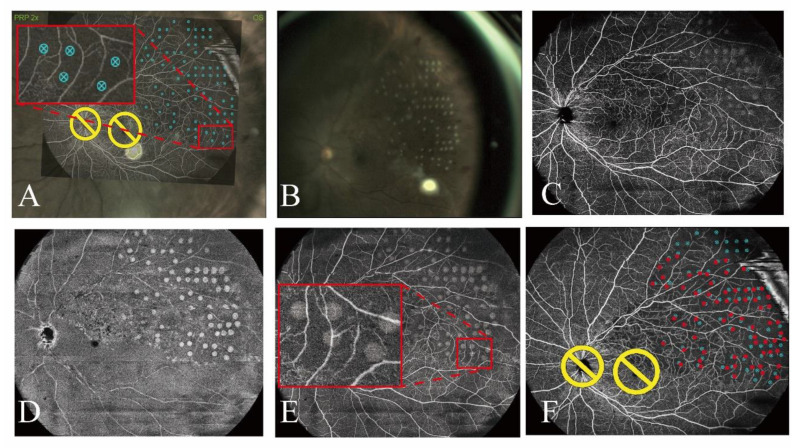
(**A**) Targeted laser spots were strategically planned to avoid collateral circulation and normal vessels, as demonstrated in the pretreatment OCTA images. (**B**) Fundus photograph after laser treatment. (**C**) OCTA image after laser treatment. (**D**) En face image after laser treatment. (**E**) The locations of the laser spots were verified on the merged OCTA and en face images. (**F**) The laser spots were marked on the OCTA images used for treatment planning. The blue and red spots indicate the targeted and actual laser spots, respectively. Yellow circles represents optic disc and macula avoided during laser treatment.

**Figure 5:**
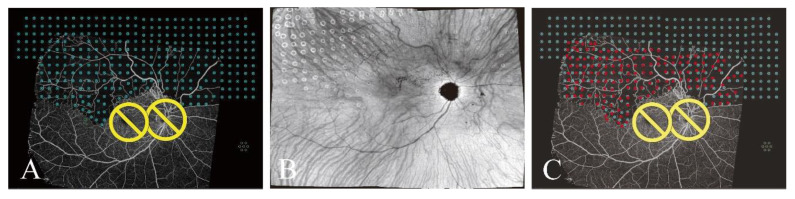
The actual positions of the spots guided by five montaged OCT images (12 × 12 mm) captured with a VG200, show minor discrepancies compared with their designated locations. (**A**) Targeted laser spots were strategically planned based on the OCTA images. (**B**) En face image after laser treatment. (**C**) The laser spots were marked on the OCTA images used for treatment planning. Blue dots indicate planned laser spots and red dots indicate the actual laser spots. Yellow circles represents optic disc and macula avoided during laser treatment.

We also investigated whether UWF-OCTA-guided navigated laser therapy could be performed while avoiding injury to collateral circulation and normal vessels in the NPAs. Targeted laser spots were strategically planned to avoid collateral circulation and normal vessels, as illustrated in the pretreatment OCTA images (Figure 4A and Figure 5A). The patients shown in Figure 4B and Figure 5B were successfully treated according to the treatment plans. However, upon merging the OCTA and en face images to verify the locations of the laser spots, we discovered that some spots were not positioned as planned, as illustrated in Figure 4A,E and Figure 5A,C. The spots within the red frames that were intended to avoid the vessels, were instead located on the vessels (Figure 4E). Further marking of the laser spots on the planned OCTA images revealed that the actual locations exhibited minor deviations from the planned locations (Figure 4F and Figure 5C).

## 4. Discussion

In this study, we provide initial evidence to show that UWF-OCTA-guided navigated laser photocoagulation is a viable therapeutic option for treating NPAs in patients with BRVO, with accurate targeting. However, the laser spots did not consistently avoid all collateral or normal vessels, as there were minor deviations between the planned and actual locations of the laser spots.

The NAVILAS^®^ navigated laser photocoagulator introduces an innovative laser approach by combining FA, color fundus images, red-free images, and infrared images with a computer-operated therapeutic 532 nm laser for precise and focused retinal therapy [26]. The UWF-OCTA images (or other images) can be obtained and imported into the NAVILAS^®^ system for offline analysis and meticulous treatment planning prior to performing the laser treatment. Notably, the NAVILAS^®^ system can superimpose these images onto live feeds, facilitating instant, targeted visualization of intended treatment zones. Unlike FA, OCTA eliminates the risk of dye leakage and offers superior axial resolution for detailed imaging of retinal capillaries. The advent of UWF-OCTA provides a unique opportunity for noninvasive assessment of retinal capillary perfusion in the posterior pole and mid-periphery [26], and mid-periphery lesions can be treated by UWF-OCTA-guided navigated laser photocoagulation. Two methods can be used to acquire UWF-OCTA images. The first utilizes a montage technique with five visual fixations (12 × 12 mm, equivalent to a FOV of 50°), achieving an ultrawide FOV of 100°–120° [27,28]. The second, newer method involves ultrahigh-speed scanning with a special lens that can capture similar UWF images (FOV of 100°–120°) in a single 23–24 × 20 mm scan [27]. In our study, UWF-OCTA images obtained using both methods were successfully transferred into the NAVILAS^®^ system without registration errors, suggesting that these images could accurately guide the treatment of NPAs.

Hypoxia increases the expression of VEGF, a potent inducer of vascular permeability that causes ME [29]. Although intravitreal anti-VEGF therapy rapidly ameliorates macular thickening, repeated injections are often necessary to sustain the initial effect. The photocoagulation of retinal NPAs in patients with ME secondary to BRVO can enhance the effects of anti-VEGF therapy on ME and reduce the frequency of anti-VEGF injections [7,8,9]. However, conventional pattern lasers, which rely on the operator’s experience, cannot accurately target NPAs (Figure 2). The mistaken identification of NPAs by conventional lasers may lead to inadequate photocoagulation and excessive treatment, potentially injuring the normal retina and exacerbating damage to peripheral visual fields. Studies have shown that OCTA can more accurately detect NPAs in BRVO [14]. In the present study, we successfully used UWF-OCTA-guided navigated lasers to treat areas missed by previous conventional laser therapy, and we found that UWF-OCTA guidance feasibly treated the NPAs in patients with previously untreated BRVO. These results suggest that UWF-OCTA-guided laser photocoagulation can feasibly treat NPAs, potentially overcoming the limitations of conventional laser systems.

Collateral vessels are frequently observed in BRVOs and can be observed in the acute phase at the earliest [12,30]. Animal studies have shown that collateral vessels appear within 3–5 days of the onset of BRVO [31,32]. Collateral vessels are considered to be distinct from retinal neovascularization [33] because they rarely leak [32,34,35]. The mean reduction in central retinal thickness was significantly greater in eyes with collateral vessels than in eyes without [11,12]. It seems that collateral vessels assisted with drainage of the obstructed venous flow into the non-obstructed areas and decreased the retinal venous pressure and vascular permeability, leading to spontaneous resolution of macular edema [34]. Im et al. [30] reported that accidental laser photocoagulation of collateral vessels aggravated leakage in eyes with BRVO, suggesting a risk of collateral vessel occlusion and subsequent worsening of ME. Therefore, careful and precise laser photocoagulation is necessary to avoid damaging the collateral vessels. In this study, we used OCTA images, which have been reported to better visualize the collateral vessels than FA images in eyes with BRVO [15], to guide laser treatment using the NAVILAS^®^ system. Unexpectedly, we found that not all of the laser spots avoided the collateral or normal vessels, as they did not match the planned positions precisely. This suggests that, while the use of UWF-OCTA-guided navigated laser improves the treatment accuracy compared with manual therapy, this approach may not achieve the expected level of precision. This problem might arise from the 3D structure of the eye and the fact that OCTA imaging requires conversion of the 3D structure into a 2D image. Fundus photography provides direct en face imaging, whereas OCTA requires reconstruction from sequential B-scans. Variations in algorithms introduce minor spatial discrepancies, especially in mid-periphery where the curvature is greater. Consequently, using a 2D OCTA image to navigate a 3D retinal structure introduces deviations from the actual positions. Additionally, errors may increase toward the periphery due to the steeper curvature. Notably, our laser therapy deliberately avoided the macular region. When conducting laser treatment on the macular area, clinicians must recognize that even minor positional deviations could induce irreversible central vision loss due to the particularity of the anatomical structure of this area.

One limitation of this study is its retrospective design; the absence of a control group or comparator group limits the ability to evaluate the effectiveness of UWF-OCTA-guided laser therapy compared to other imaging modalities or treatment strategies. Additionally, UWF-OCTA can only image NPAs in the mid-periphery. Moreover, we did not investigate the impact of precise laser treatment of NPAs on ME resolution and visual field preservation. These aspects will be addressed in future studies. Furthermore, as en face OCTA images are two-dimensional projections of a three-dimensional retinal surface, any observed deviation between planned and actual laser spot locations reflects projection-related artifacts rather than true spatial displacements. Consequently, a quantitative assessment of these deviations is not reliable without appropriate 3D curvature correction. Future studies should explore advanced image registration algorithms to enable accurate quantification of positional deviations in navigated laser treatments.

## 5. Conclusions

This study represents an early feasibility assessment of UWF-OCTA-guided navigated laser photocoagulation using a NAVILAS^®^ system for treating NPAs in patients with BRVO. However, minor deviations were observed between the planned and actual locations, attributable to the inherent challenges of using two-dimensional (2D) UWF-OCTA images to guide treatments on three-dimensional (3D) retinal structures. This discrepancy should be carefully considered when using OCTA-guided navigated laser photocoagulation, particularly in the treatment of macular area diseases. Future studies should incorporate 3D curvature correction models or AI-guided registration algorithms to mitigate projection errors and potentially enhance therapeutic outcomes.

## Figures and Tables

**Table 1:** Success rate of UWF-OCTA images obtained using different devices to guide navigated laser treatment of NPAs.

	VG200	Xephilio OCT-S1	Bmizar	*p* *
No. of Patients	10	10	8	N/A
No. of Successful Treatments	10	8	8	N/A
Success Rate	100%	100%	100%	N/A

* If the success rate is identical at 100% in all groups, there is no basis for comparison using a χ^2^ test. N/A, not applicable; NPA, non-perfused area; UWF-OCTA, ultrawide-field optical coherence tomography.

## Data Availability

The original contributions presented in this study are included in the article. Further inquiries can be directed to the corresponding authors.

## Abbreviations

The following abbreviations are used in this manuscript:

UWF-OCTA	ultrawide-field optical coherence tomography angiography
NPAs	non-perfused areas
BRVO	branch retinal vein occlusion
ME	macular edema
VEGF	anti-vascular endothelial growth factor
FA	fluorescein angiography
FOV	field of view

## References

[1] Jonas J., Paques M., Monés J., Glacet-Bernard A. (2010). Retinal vein occlusions. Dev. Ophthalmol..

[2] Wong T.Y., Scott I.U. (2010). Retinal-Vein Occlusion. N. Engl. J. Med..

[3] Campochiaro P.A., Hafiz G., Shah S.M., Nguyen Q.D., Ying H., Do D.V., Quinlan E., Zimmer-Galler I., Haller J.A., Solomon S.D. (2008). Ranibizumab for Macular Edema Due to Retinal Vein Occlusions: Implication of VEGF as a Critical Stimulator. Mol. Ther..

[4] Campochiaro P.A., Sophie R., Pearlman J., Brown D.M., Boyer D.S., Heier J.S., Marcus D.M., Feiner L., Patel A. (2014). Long-term Outcomes in Patients with Retinal Vein Occlusion Treated with Ranibizumab. Ophthalmology.

[5] Hayreh S.S., Rubenstein L., Podhajsky P. (1993). Argon Laser Scatter Photocoagulation in Treatment of Branch Retinal Vein Occlusion. Ophthalmologica.

[6] Branch Vein Occlusion Study Group (1986). Argon laser scatter photocoagulation for prevention of neovascularization and vitreous hemorrhage in branch vein occlusion. Arch Ophthalmol..

[7] Tomomatsu Y., Tomomatsu T., Takamura Y., Gozawa M., Arimura S., Takihara Y., Inatani M. (2015). Comparative study of combined bevacizumab/targeted photocoagulation vs bevacizumab alone for macular oedema in ischaemic branch retinal vein occlusions. Acta Ophthalmol..

[8] Ravani R.D., Goel S., Kumar A., Chandra P., Chandra M., Kumar V. (2019). Comparison of ranibizumab alone versus ranibizumab with targeted retinal laser for branch retinal vein occlusion with macular edema. Indian J. Ophthalmol..

[9] An S.H., Jeong W.J. (2019). Early-scatter laser photocoagulation promotes the formation of collateral vessels in branch retinal vein occlusion. Eur. J. Ophthalmol..

[10] Terashima H., Okamoto F., Hasebe H., Ueda E., Yoshida H., Fukuchi T. (2021). Optical coherence tomography angiography and Humphrey field analyser for macular capillary non-perfusion evaluation in branch retinal vein occlusion. Sci. Rep..

[11] Singh R.P., Lee T.J., Yau L., Rubio R.G. (2014). Collateral vessel presence in branch and central retinal vein occlusions and their impact on visual acuity and anatomical gains. Retina.

[12] Suzuki N., Hirano Y., Tomiyasu T., Kurobe R., Yasuda Y., Esaki Y., Yasukawa T., Yoshida M., Ogura Y. (2019). Collateral vessels on optical coherence tomography angiography in eyes with branch retinal vein occlusion. Br. J. Ophthalmol..

[13] Silva P.S., Cavallerano J.D., Sun J.K., Soliman A.Z., Aiello L.M., Aiello L.P. (2013). Peripheral Lesions Identified by Mydriatic Ultrawide Field Imaging: Distribution and Potential Impact on Diabetic Retinopathy Severity. Ophthalmology.

[14] Kuehlewein L., An L., Durbin M.K., Sadda S.R. (2015). Imaging Areas of Retinal Nonperfusion in Ischemic Branch Retinal Vein Occlusion With Swept-Source OCT Microangiography. Ophthalmic Surg. Lasers Imaging Retin..

[15] Suzuki N., Hirano Y., Yoshida M., Tomiyasu T., Uemura A., Yasukawa T., Ogura Y. (2016). Microvascular Abnormalities on Optical Coherence Tomography Angiography in Macular Edema Associated With Branch Retinal Vein Occlusion. Arch. Ophthalmol..

[16] Salz D.A., de Carlo T.E., Adhi M., Moult E., Choi W., Baumal C.R., Witkin A.J., Duker J.S., Fujimoto J.G., Waheed N.K. (2016). Select Features of Diabetic Retinopathy on Swept-Source Optical Coherence Tomographic Angiography Compared With Fluorescein Angiography and Normal Eyes. JAMA Ophthalmol.

[17] Schaal K.B., Munk M.R., Wyssmueller I., Berger L.E., Zinkernagel M.S., Wolf S. (2019). Vascular Abnormalities in Diabetic Retinopathy Assessed with Swept-Source Optical Coherence Tomography Angiography Widefield Imaging. Retina.

[18] Soecknick F., Breher K., Nafar Z., Kubach S., Straub J., Wahl S., Ziemssen F. (2024). The clinical evaluation of a widefield lens to expand the field of view in optical coherence tomography (OCT-A). Sci. Rep..

[19] Gawęcki M., Kiciński K. (2024). Advantages of the Utilization of Wide-Field OCT and Wide-Field OCT Angiography in Clinical Practice. Diagnostics.

[20] Zhao X.Y., Zhao Q., Wang C.T., Meng L.H., Cheng S.Y., Gu X.W., Sadda S.R., Chen Y.X. (2024). Central and Peripheral Changes in Retinal Vein Occlusion and Fellow Eyes in Ultra-Widefield Optical Coherence Tomography Angiography. Investig. Ophthalmol. Vis. Sci..

[21] Karapapak M., Ozal E., Ermis S., Guler S., Ozal S.A. (2024). Comparative Analysis of Pain and Duration in Panretinal Photocoagulation: Navilas Laser versus Conventional Laser in Proliferative Diabetic Retinopathy. Med Bull. Sisli Hosp..

[22] Amoroso F., Pedinielli A., Cohen S.Y., Jung C., Chhablani J., Astroz P., Colantuono D., Semoun O., Capuano V., Souied E.H. (2021). Navigated micropulse laser for central serous chorioretinopathy: Efficacy, safety, and predictive factors of treatment response. Eur. J. Ophthalmol..

[23] Laich Y., Farassat N., Grewing V., Boehringer D., Bucher F., Maloca P.M., Reinhard T., Lang S.J., Agostini H., Reich M. (2024). Optical Coherence Tomography Angiography–Navigated Laser Photocoagulation of Retinal Hemangioblastomas in Patients With von Hippel–Lindau Disease. Transl. Vis. Sci. Technol..

[24] Amoroso F., Souied E.H., Cohen S.Y., Pedinielli A., Astroz P., Garavito R.B., Capuano V., Querques G., Miere A. (2020). OCTA-guided navigated laser therapy for advanced macula neovascularization secondary to age related macular degeneration. Eur. J. Ophthalmol..

[25] Schmidt-Erfurth U., Garcia-Arumi J., Gerendas B.S., Midena E., Sivaprasad S., Tadayoni R., Wolf S., Loewenstein A. (2019). Guidelines for the Management of Retinal Vein Occlusion by the European Society of Retina Specialists (EURETINA). Ophthalmologica.

[26] Kernt M., Cheuteu R., Vounotrypidis E., Haritoglou C., Kampik A., Ulbig M.W., Neubauer A.S. (2010). Focal and panretinal photocoagulation with a navigated laser (NAVILAS^®^). Acta Ophthalmol..

[27] Kalra G., Pichi F., Menia N.K., Shroff D., Phasukkijwatana N., Aggarwal K., Agarwal A. (2021). Recent advances in wide field and ultrawide field optical coherence tomography angiography in retinochoroidal pathologies. Expert Rev. Med Devices.

[28] Sakimoto S., Kawasaki R., Nishida K. (2020). Retinal Neovascularization–Simulating Retinal Capillary Reperfusion in Branch Retinal Vein Occlusion, Imaged by Wide-Field Optical Coherence Tomography Angiography. JAMA Ophthalmol..

[29] Liberski S., Wichrowska M., Kocięcki J. (2022). Aflibercept versus Faricimab in the Treatment of Neovascular Age-Related Macular Degeneration and Diabetic Macular Edema: A Review. Int. J. Mol. Sci..

[30] Im C.Y., Lee S.Y., Kwon O.W. (2002). Collateral vessels in branch retinal vein occlusion. Korean J. Ophthalmol..

[31] Genevois O., Paques M., Simonutti M., Sercombe R., Seylaz J., Gaudric A., Brouland J.-P., Sahel J., Vicaut E. (2004). Microvascular Remodeling after Occlusion-Recanalization of a Branch Retinal Vein in Rats. Investig. Opthalmol. Vis. Sci..

[32] Klein R., Klein B., Henkind P., Bellhorn R. (1971). Retinal collateral vessel formation. Investig. Ophthalmol..

[33] Henkind P., Wise G.N. (1974). Retinal neovascularization, collaterals, and vascular shunts. Br. J. Ophthalmol..

[34] Christoffersen N.L., Larsen M. (1999). Pathophysiology and hemodynamics of branch retinal vein occlusion. Ophthalmology.

[35] Weinberg D.V., Wahle A.E., Ip M.S., Scott I.U., VanVeldhuisen P.C., Blodi B.A. (2013). Score Study Report 12: Development of venous col-laterals in the Score Study. Retina.

